# Nitrate of Strichnine in Paralysis, in a Letter to the Editor

**Published:** 1834

**Authors:** Edward Ellis

**Affiliations:** Meadville, Pa.


### Art. II.—Nitrate of Strichnine in Paralysis.

*A short account of the beneficial effects of the Nitrate of Strichnine in Paralysis, in a letter to the Editor.*

*Meadville, Pa., Feb. 20th*, 1834.

Sir,

I take the liberty of communicating to you, in a brief sketch, of the result of my experience, with the *Nitrate* of Strichnine, in the cure of Paralysis.

This form, or preparation of Strichnine, is to me a new one, as a medicine. The disagreeable symptoms resulting from the use of the Nex Vomica, or Strichnine, has prevented my paying much attention, to any of the other forms.

Dr. Kruse, a German Chemist, now residing in this place, having made a very beautiful preparation of this article, I was induced to try it, and I am happy to say, it more than answered my most sanguine expectations.

My first trial of this medicine, was in the case of a young man, 24 years of age, who had been laboring under severe Scrofula, for about three years; for which he had been under medical advice during a year's residence in Baltimore, and about the same length of time in Pittsburgh, with little or no relief. His case was pronounced incurable; and as a last resort, he was directed to use Swaim's Panacea.

When he had taken about half the contents of a second bottle, Ptyalism was produced, attended with great debility of the whole system. The ulcers became very painful, with coldness and pain in the extremities, which increased until he lost all power of motion. The Panacea was then abandoned.

He remained in this helpless condition about two months, when he applied to me for relief from his severe sufferings, though he had given up all hope of recovery.

His pulse was frequent—small and somewhat wirey—respiration hurried—skin smooth and glossy, but little or no swelling underneath—tongue clean. He had a large ulcer directly over the sternum, about six inches in the greatest diameter, and four in the least, discharging a bloody sanious matter. Another from the right axilla, opening backward, near the edge of the scapula, which also presented the same unhealthy appearance. The left axillary gland produced a tumor about four or five inches in diameter, presenting a hard, uneven surface, very painful.

My first attention was directed to the scrofulous disease, which entirely disappeared in less than six weeks. (The course I have pursued in the treatment of scrofula, for two years past, I intend to present to you at another time.) Unfortunately, however, he remained as helpless as ever. After making several trials of the usual remedies, which proved fruitless, (as I believe they too often do, in cases of long standing,) I commenced administering the Nitrate of Strichnine, in form of solution. (Nit. Strich. i. gr. Dil. Alcoh. ℨij mix.) Immediately after taking one drachm of this solution, he felt a glow in every part of his system, which continued about three hours, when his former disagreeable sensations began to return. The same quantity was again administered, with the same good effect, and repeated every third hour, increasing one drachm of the solution daily. On the fourth day he was able to feed himself; and at the end of four weeks, he walked across his room with no other assistance than his cane—entirely free from pain—all the functions of the system performing their offices in a healthy manner. In a case of Paraplegia, I was equally successful.

So far as my experience will permit me to judge, I consider this form of Strichnine, as decidedly preferable to any other; as being mild in its operation, and much more safe than either the Nex Vomica or Strichinine. It produces no irritation of the stomach, and instead of exciting spasms, it entirely allays the spasmodic twitchings, often so distressing to paralytic patients.

It was not my intention to give a labored essay on this subject, but merely to express, in a brief manner, the result of my practice in the use of this medicine, hoping it may be a means of aiding some of my professional brethren, in giving relief to suffering humanity.

I will merely add, that much care is necessary in the preparation of this article, the crystals should form a white, pearly needles—not a brown dust as we often find it in the shops.

Should you find any thing in the foregoing statement worthy of notice, you are at liberty to make such use of it as you think proper.

Yours respectfully,

EDWARD ELLIS, M. D.

Dr. Drake.

